# Supplementary material for: A scalable implementation of the recursive least-squares algorithm for training spiking neural networks
Source: Front Neuroinform. 2023 Jun 27;17:1099510. doi: 10.3389/fninf.2023.1099510 (PMC10333503; doi:10.3389/fninf.2023.1099510)
Supplement: Supplementary file 1 [file Presentation_1.pdf]

4 Appendix

Here we provide technical details on how spiking neural networks were trained. A large part of following presentation was adopted from the previous paper of the authors [9].

4.1 Generating target neural trajectories from PSTHs

For each recorded neuron, we converted its PSTH to target pre-synaptic activities to be used for training the synaptic inputs. Specifically, in Figure 2 we obtained for each spike rate r_{it} ($i = 1, ..., N$, and $t = T_{init} + \Delta t, ..., T_{end}$ where $T_{init} = -2$ and $T_{end} = 1$) the mean synaptic input f_{it} that needs to be applied to the the leaky integrate-and-fire neuron to generate the desired spike rate. To this end, we numerically solved the nonlinear rate equation

$$r_{it} = \phi(f_{it}, \sigma^2) \tag{1}$$

where

$$\phi(m, \sigma) = \tau_m^{-1}[\sqrt{\pi}\int_{\frac{V_{reset}-m}{\sigma}}^{\frac{V_{thr}-m}{\sigma}} dw e^{w^2} \mathrm{erfc}(-w)]^{-1}$$

is the transfer function of the leaky integrate-and-fire neuron given mean input, m, and variance of the input, σ^2 [38, 28]. This conversion yielded a set of vectors of target synaptic inputs $\mathbf{f}_1, ..., \mathbf{f}_N \in \mathbb{R}^T$ where $T = (T_{end} - T_{init})/\Delta t$ for the recorded neurons. Note that in Figure 2 we did not simulate the recurrent static connections ($K = 0$). Simulating both static connections and the external noise would require estimating σ in Eq.(1). This can be done by assuming Poisson firing statistics and calculating σ given the average connectivity and rates of the populations in the network [28]. Alternatively, it is possible to directly estimate σ from simulating an initial network that fits the average rates and level of noise (e.g. the Fano factor) observed in the data [9]. The results presented in this paper are done using the latter approach.

4.2 Network connectivity

In Figure 1A, the spiking neural network consisted of randomly connected N_E excitatory and N_I inhibitory neurons. They were recurrently connected as in [9]. In short, the recurrent synapses consisted of static weights $\mathbf{J}$ that remained constant throughout training and plastic weights $\mathbf{W}$ that were modified by the training algorithm. The static synapses connected neuron j in population β to neuron i in population α with probability $p_{\alpha\beta} = K_{\alpha\beta}/N_\beta$ and synaptic weight $\bar{J}_{\alpha\beta}/\sqrt{K_{\alpha\beta}}$, where $K_{\alpha\beta}$ is the average number of static connections from population β to α:

$$Pr(J_{ij}^{\alpha\beta} \neq 0) = \frac{K_{\alpha\beta}}{N_\beta}. \tag{2}$$

The strength of plastic synapses, $\bar{W}_{\alpha\beta}/\sqrt{K_{\alpha\beta}}$, was of the same order as the static weights. However, the plastic synapses connected neurons with a smaller probability:

$$Pr(W_{ij}^{\alpha\beta} \neq 0) = \frac{L_{\alpha\beta}}{N_\beta} \quad \text{with} \quad L_{\alpha\beta} = c\sqrt{K_{\alpha\beta}} \tag{3}$$

which made the plastic synapses much sparser than the static synapses [39, 9]. Here, c is an order 1 parameter that depends on training setup.

The static and plastic connections were non-overlapping in that any two neurons in the network can have only one type of synapse.

$$J_{ij}^{\alpha\beta} W_{ij}^{\alpha\beta} = 0. \tag{4}$$

Keeping them disjoint allowed us to maintain the initial network dynamics generated by the static synapses and, subsequently, introduce trained activity to the initial dynamics by modifying the plastic synapses.

The static recurrent synapses were strong in that the coupling strength between two connected neurons scaled as $\frac{1}{\sqrt{K_{\alpha\beta}}}$, while the average number of synaptic inputs scaled as $K_{\alpha\beta}$. As a result of this strong scaling, the excitatory and inhibitory synaptic inputs to a neuron from static synapses increased as $\sqrt{K_{\alpha\beta}}$, and thus were much larger than the spike-threshold for a large $K_{\alpha\beta}$. However, the excitatory and inhibitory currents were dynamically canceled, and, together with the external input, the sum was balanced to be around the spike-threshold [20, 9].

In contrast to the static synapses, each trained neuron received only about $L = \sqrt{K_{\alpha\beta}}$ plastic synapses. This made the plastic synapses much sparser than the sparse static EI connectivity (e.g., with $K = 1000$ static synapses, there are of the order of $\sqrt{K} \approx 30$ plastic synapses per neuron). Consequently, the EI plastic inputs of the initial network were independent of $K_{\alpha\beta}$ and substantially weaker than the EI balanced inputs for a large $K_{\alpha\beta}$. After training the plastic synapses, the total synaptic input, i.e., the sum of plastic and balanced inputs, to each trained neuron was able to follow the target patterns. With this scaling of plastic synapses, training was robust to variations in the network size, N, and the number of pre-synaptic connections, $K_{\alpha\beta}$ (Fig. 1B).

In Figures 1C, E and Figure 2, on the other hand, the network had only plastic synapses but no static synapses, i.e., $K = 0$. Without the static excitatory-inhibitory synaptic connections, as in Figure 1, the network lacked internally generated noise. Therefore, we injected external noise to the neuron's voltage equation to induce variability in spiking activity (Fig. 2C). The variance of this white noise, σ^2, was chosen such that the externally injected noise was similar to internally generated fluctuating synaptic current when static connections were present ($K = 1000$).

4.3 Network dynamics

In the following mathematical description of network activity, the static connections present in Figure 1B ($K > 0$) produced the balanced inputs ($u_{bal,i}^{\alpha}$), which exhibited large fluctuations. For this reason, no additional external noise was injected to the network ($\sigma = 0$). On the other hand, as mentioned above, in Figure 2 (and also in Figs. 1C, E), the static connections did not exist ($K = 0$) and external noise was injected to the neurons ($\sigma > 0$).

We used an integrate-and-fire neuron to model the membrane potential dynamics of i'th neuron:

$$\tau_m \dot{v}_i^{\alpha} = -v_i^{\alpha} + u_i^{\alpha} + X_i^{\alpha} + \sigma \xi_i \tag{5}$$

where a spike is emitted and the membrane potential is reset to v_{reset} when the membrane potential crosses the spike-threshold v_{thr}. The membrane equations (Eq. 5), and the following synaptic equations (Eq. 8), were numerically solved using the forward Euler method.

Here, u_i^{α} is the total synaptic input to neuron i in population α that can be divided into static and plastic inputs incoming through the static and plastic synapses, respectively:

$$u_i^{\alpha} = u_{bal,i}^{\alpha} + u_{plas,i}^{\alpha}. \tag{6}$$

X_i^α is the total external input that can be divided into constant external input, plastic external input, and the stimulus:

$$X_i^\alpha = X_{bal,i}^\alpha + X_{plas,i}^\alpha + X_{stim,i}^\alpha. \tag{7}$$

$X_{bal,i}^\alpha$ is a constant input associated with the initial balanced network. It scales with the number of connections (i.e. is proportional to $\sqrt{K_{\alpha\beta}}$), determines the firing rate of the initial network and stays unchanged [20]. $X_{plas,i}^\alpha$ is plastic input provided to trained neurons in the recurrent network from external neurons that emit stochastic spikes with pre-determined rate patterns. The synaptic weights from the external neurons to the trained neurons were modified by the training algorithm. $X_{stim,i}^\alpha$ is the pre-determined stimulus, generated independently from the Ornstein-Uhlenbeck process for each neuron, and injected to all neurons in the network to trigger the learned responses in the trained neurons.

The synaptic activity was modeled by an instantaneous jump of the synaptic input due to a presynaptic neuron's spike, followed by an exponential decay. Our network did not include time delays in the propagation of the presynaptic neuron's spike to the post-synaptic neuron; therefore a spike caused jumps in the postsynaptic neurons instantaneously. Since the static and plastic synapses did not overlap, we separated the total synaptic input into static and plastic components as mentioned above:

$$\begin{aligned} \tau_{bal}\dot{u}_{bal,i}^\alpha &= -u_{bal,i}^\alpha + \sum_{\beta\in\{E,I\}}\sum_{j\in\beta} J_{ij}^{\alpha\beta}\sum_{t_k^j<t}\delta(t-t_k^j) \\ \tau_{plas}\dot{u}_{plas,i}^\alpha &= -u_{plas,i}^\alpha + \sum_{\beta\in\{E,I\}}\sum_{j\in\beta} W_{ij}^{\alpha\beta}\sum_{t_k^j<t}\delta(t-t_k^j). \end{aligned} \tag{8}$$

Alternatively, the synaptic activity can be expressed as a weighted sum of filtered spike trains because the synaptic variable equations (Eq. 8) are linear in $\mathbf{J}$ and $\mathbf{W}$:

$$\begin{aligned} u_{bal,i}^\alpha &= \sum_{\beta,j} J_{ij}^{\alpha\beta} r_{bal,j}^\beta \\ u_{plas,i}^\alpha &= \sum_{\beta,j} W_{ij}^{\alpha\beta} r_{plas,j}^\beta \end{aligned} \tag{9}$$

where

$$\begin{aligned} \tau_{bal}\dot{r}_{bal,i}^\beta &= -r_{bal,i}^\beta + \sum_{t_k^i<t}\delta(t-t_k^i) \\ \tau_{plas}\dot{r}_{plas,i}^\beta &= -r_{plas,i}^\beta + \sum_{t_k^i<t}\delta(t-t_k^i) \end{aligned} \tag{10}$$

describe the dynamics of synaptically filtered spike trains.

Each external neuron emitted spikes stochastically at a pre-defined rate that changed over time. The rate patterns, followed by the external neurons, were randomly generated from an Ornstein-Uhlenbeck process with mean rate of 5 Hz. The synaptically filtered external spikes were weighted by plastic synapses $\mathbf{W_X}$ and injected to trained neurons:

$$X_{plas,i}^\alpha = \sum_j W_{ij}^{\alpha X} r_j^X \tag{11}$$

where

$$\tau_{plas}\dot{r}_{plas,i}^X = -r_{plas,i}^X + \sum_{t_k^i<t}\delta(t-t_k^i) \tag{12}$$

Similarly, the external stimulus $X_{stim,i}$ applied to each neuron i in the network to trigger the learned response is generated independently from the Ornstein-Uhlenbeck process.

In the following section, we will use the linearity of $\mathbf{W}, \mathbf{W_X}$ in Eqs. 9 and 11 to derive the training algorithm that modifies plastic synaptic weights.

4.4 Recursive least squares

Here we derive a synaptic update rule that modifies the plastic synapses to learn the target activities. The derivation closely follows previous papers [8, 16, 9]. For notational simplicity, we drop the neuron index i in $\mathbf{w}_i$ and other variables (e.g., f_i, u_i) but the same synaptic update rule is applied to all trained neurons. In fact, the plastic connections to every trained neuron can be updated simultaneously since each trained neuron has its own private target function and plastic connections. Therefore, the CPU multithreading (Fig. 1D) or GPU implementation (Figs. 1B, C) presented in our study leverages the fact that the synaptic update rule can be applied in parallel to all plastic synapses.

The cost function of each trained neuron i is defined by

$$C[\mathbf{w}] = \frac{1}{2}\sum_t (f_t - u_t)^2 + \lambda\|\mathbf{w}\|^2 + \mu\Big((\mathbf{w}\cdot\mathbf{1}_E)^2 + (\mathbf{w}\cdot\mathbf{1}_I)^2\Big) \tag{13}$$

Specifically, $\mathbf{w} = (W_{i_1}, ..., W_{i_L})$ is a vector of recurrent plastic presynaptic synapses indexed by $i_1, ..., i_L$; f_t is the target function over time t; u_t is the total synaptic input over time t. The hyperparameters λ and μ are the standard L_2 and ROWSUM [16] regularization terms, respectively. The L_2 regularization controls the overall learning rate, and ROWSUM regularization constrains the strength aggregate excitatory and inhibitory synaptic weights to the trained neuron.

The gradient of the cost function with respect to the weights $\mathbf{w}$ is

$$\begin{aligned}\nabla_{\mathbf{w}} C &= \frac{1}{2}\nabla_{\mathbf{w}}\Big[\sum_t (f_t - u_t)^2 + \lambda\|\mathbf{w}\|^2 + \mu\Big((\mathbf{w}\cdot\mathbf{1}_E)^2 + (\mathbf{w}\cdot\mathbf{1}_I)^2\Big)\Big] \\ &= \sum_t (-f_t\mathbf{r}_t + \mathbf{r}_t\mathbf{r}_t'\mathbf{w}) + \lambda\mathbf{w} + \mu(\mathbf{1}_E\mathbf{1}_E' + \mathbf{1}_I\mathbf{1}_I')\mathbf{w}\end{aligned} \tag{14}$$

where we substitute the expression $u_t = \mathbf{w}\cdot\mathbf{r}_t$ in the first line to evaluate the gradient with respect to $\mathbf{w}$. To derive the synaptic update rule, we compute the gradient at two consecutive time points

$$\mathbf{0} = \nabla_{\mathbf{w}_n} C = \sum_{t=1}^{n} (-f_t\mathbf{r}_t + \mathbf{r}_t\mathbf{r}_t'\mathbf{w}_n) + \lambda\mathbf{w}_n + \mu(\mathbf{1}_E\mathbf{1}_E' + \mathbf{1}_I\mathbf{1}_I')\mathbf{w}_n \tag{15}$$

and

$$\mathbf{0} = \nabla_{\mathbf{w}_{n-1}} C = \sum_{t=1}^{n-1} (-f_t\mathbf{r}_t + \mathbf{r}_t\mathbf{r}_t'\mathbf{w}_{n-1}) + \lambda\mathbf{w}_{n-1} + \mu(\mathbf{1}_E\mathbf{1}_E' + \mathbf{1}_I\mathbf{1}_I')\mathbf{w}_{n-1}. \tag{16}$$

Subtracting Eqs (15) and (16) yields

$$\begin{aligned}\mathbf{w}_n &= \mathbf{w}_{n-1} + e_n P_n \mathbf{r}_n \\ e_n &= f_n - \mathbf{w}_{n-1}\cdot\mathbf{r}_n\end{aligned} \tag{17}$$

where

$$P^n = \Big[\sum_{t=1}^{n} \mathbf{r}_t(\mathbf{r}_t)' + \lambda I + \mu\mathbf{1}_E\mathbf{1}_E' + \mu\mathbf{1}_I\mathbf{1}_I'\Big]^{-1} \quad \text{for} \quad n \geq 1 \tag{18}$$

with the initial value

$$P^0 = [\lambda I + \mu \mathbf{1}_E \mathbf{1}_E' + \mu \mathbf{1}_I \mathbf{1}_I']^{-1}. \tag{19}$$

To update P^n iteratively, we use the Woodbury matrix identity

$$(A + UCV)^{-1} = A^{-1} - A^{-1}U(C^{-1} + VA^{-1}U)^{-1}VA^{-1} \tag{20}$$

where A is invertible and $N \times N$, U is $N \times T$, C is invertible and $T \times T$ and V is $T \times N$ matrices. Then P^n can be calculated iteratively

$$P^n = P^{n-1} - \frac{P^{n-1}\mathbf{r}_n(\mathbf{r}_n)'P^{n-1}}{1 + (\mathbf{r}_n)'P^{n-1}\mathbf{r}_n}. \tag{21}$$

The following pseudocode provides overview of the simulation and RLS training of spiking neural networks.

Algorithm 1 Simulation of spiking neural networks with RLS training

```
for t = 1 : Nstep do                                                    ▷ Iterate over time steps
    Initialize: inputs, inputsPlastic, spikes = 0

    if training and mod(t, learnEvery) = 0 then   ▷ If training, learn weights every learnEvery
        wpWeightOut = RLS(r, P, Px, wpWeightIn, u[c], X[c], utarg)
    end if

    for c = 1 : Ncells do                                               ▷ Update cell activities
        u[c] += −dt · u[c]/τ_syn + inputs/τ_syn                           ▷ Update static input
        u_plas[c] += −dt · u_plas[c]/τ_plas + inputsPlastic/τ_plas        ▷ Update plastic input
        r[c] += −dt · r[c]/τ_plas + spikes[c]/τ_plas             ▷ Filtered spikes to be used in RLS
        u_total[c] = u[c] + u_plas[c]

        if stimulus on < t < stimulus off then                          ▷ Apply external stimulus
            X[c] = Xbal[c] + Xstim[c]        ▷ External stimulus to trigger trained activity patterns
        else
            X[c] = Xbal[c]                                              ▷ Baseline external stimulus
        end if

        v[c] += dt/τ_mem · (−v[c] + X[c] + u_total[c])                  ▷ Update membrane potential
        if v[c] > spike threshold then                                  ▷ Emit spike
            spikes[c] = 1                                  ▷ Propagate spikes to postsyn. neurons
            for j = 1 : nc0[c] do                                       ▷ via static synapses
                wgt = w0Weight[j, c]
                post_cell = w0Index[j, c]
                inputs[post_cell] += wgt
            end for
            for j = 1 : ncpOut[c] do                                    ▷ via plastic synapses
                wgt_plas = wpWeightOut[j, c]
                post_cell = wpIndex[j, c]
                inputsPlastic[post_cell] += wgt_plas
            end for
        end if
    end for
end for
```

Algorithm 2 Recursive least squares algorithm

```
function RLS(r,P,Px,wpWeightIn,u[c],X[c],utarg)
    r_pre = r[Px[c]]                        ▷ Spiking activity of neurons presynaptic to neuron c
    P[c] += −P[c] r_pre r'_pre P[c]/(1 + r'_pre P[c] r_pre)
    e = wpWeightIn[c, :] · r_pre + u[c] + X[c] − utarg[c, t]                    ▷ Learning error
    dw = −e P[c] r_pre
    wpWeightIn[c, :] += dw                                                      ▷ Update weights
    Convert wpWeightIn to wpWeightOut        ▷ Convert matrix format to ease following simulation
    return wpWeightOut
end function
```
